# Exploring factors influencing university students’ entrepreneurial intentions: The role of attitudes, beliefs, and environmental support

**DOI:** 10.1371/journal.pone.0316392

**Published:** 2025-01-09

**Authors:** Qing Liu, Michael Yao-Ping Peng

**Affiliations:** 1 Hunan University of International Economics, Changsha, China; 2 School of Management, Foshan University, Foshan, China; Sichuan Agricultural University, CHINA

## Abstract

Entrepreneurship is an increasingly popular career choice among students, driven by the transformative impact of emerging technologies and evolving professional landscapes. This study focuses on how higher education shapes students’ professional identities and entrepreneurial intentions, particularly among business school students. Utilizing the Theory of Planned Behavior (TPB) as the foundational framework, the study examines the factors influencing entrepreneurial intentions, with a specific emphasis on the moderating role of departmental identification. The primary aim of this research is to explore how students’ identification with their academic departments influences the relationship between entrepreneurial attitude, self-efficacy, and environmental support with entrepreneurial intentions. The study hypothesizes that the stronger the departmental identification, the more significant these relationships become. A survey is conducted among students from several public universities in the eastern provinces of Mainland China, yielding 1,632 valid responses. The results confirm a positive correlation between entrepreneurial attitude, self-efficacy, and environmental support with entrepreneurial intentions. Furthermore, departmental identification moderates these relationships, amplifying the effects when students identify strongly with their academic departments. These findings emphasize the critical role of departmental identity in shaping entrepreneurial aspirations. They highlight the need for higher education institutions to leverage departmental identity as a strategic tool to guide students’ career trajectories. By fostering a supportive academic environment that strengthens departmental identity, institutions can better prepare their students for entrepreneurial success in a rapidly evolving professional world.

## 1. Introduction

Higher education is a crucial phase in shaping students’ social identities, as the career paths of many individuals are often determined after completing their university studies. Recent studies highlight the significance of curricula and academic environments in consolidating students’ professional identities, which play a critical role in determining their career choices [1, 2]. Students’ identification with their departments influences not only their level of engagement and career attitudes but also their academic and social interactions, and their willingness to continue their education or work in their department [3, 4]. Despite its well-recognized importance in Western education, professional identity’s influence on entrepreneurial intentions remains underexplored in Asian contexts, necessitating further investigation [5]. This study aims to address this gap by examining how higher education fosters students’ professional identities, particularly their entrepreneurial aspirations, among business school students in Asia.

The application of knowledge gained during studies is a key expectation of education. However, several factors influence graduates’ likelihood of selecting an industry related to their field of study as their first job. Consequently, entrepreneurship has emerged as a significant career option for university graduates, particularly after completing higher education [6]. With the increasing prevalence of e-commerce and emerging technologies, research interest in student entrepreneurship has grown [7–9]. Management, marketing, finance, and other relevant courses equip students with the necessary professional competencies for entrepreneurship [10]. Given the professional competencies provided by business school curricula, this study focuses on how management education strengthens entrepreneurial intentions through specialized knowledge and professional identity.

Numerous studies focus on business school students’ entrepreneurial intentions due to their educational background. For example, Krueger et al. [11] and Amofah et al. [12] analyze entrepreneurial intentions among business school students. Despite these studies, research on the influence of professional identity on entrepreneurial intentions is scarce [13]. Obschonka et al. [14] used the Theory of Planned Behavior (TPB) to explore the effect of group identification on entrepreneurial intentions. The study revealed that group identification might moderate the relationship between entrepreneurial intentions and socio-environmental factors and self-perceived abilities. Specifically, individuals with higher group identification were more strongly influenced by entrepreneurial attitudes, socio-environmental factors, and self-perceived abilities. This suggests that the strength of group identification amplifies the impact of these factors on entrepreneurial intentions, highlighting the complex interplay between personal and environmental influences. In considering the moderating role of identity, this study chooses to focus on departmental identification rather than university-wide identification. Departmental identification is considered more relevant to students’ academic and professional experiences because the specialized knowledge and skills provided by their departments are directly applicable to their entrepreneurial development. While university-wide identity can also be significant, the department-specific focus allows for a more precise analysis of the immediate academic context that influences entrepreneurial intentions. Therefore, this study aims to explore how students’ identification with their departments influences their entrepreneurial intentions.

While most studies on the factors influencing entrepreneurial intentions among university students have focused on business school students, only a handful have investigated the role of entrepreneurial self-efficacy. Entrepreneurial self-efficacy, defined as an individual’s belief in their ability to successfully perform the tasks required to start and manage a business [15], significantly influences one’s confidence in undertaking entrepreneurial activities and overcoming challenges [16]. Higher levels of entrepreneurial self-efficacy are linked to greater entrepreneurial intentions, as individuals who believe in their entrepreneurial capabilities are more likely to pursue and persist in entrepreneurial endeavors [17]. Some studies have found significant differences in entrepreneurial intentions and competencies among students from different departments, possibly due to varying emphases on professional competencies in departmental curricula [18, 19]. Emerging technologies like e-commerce and AI have led many business schools to rethink their curricula, creating pressure to revise educational goals and programs. Offering complementary entrepreneurship education can provide students with greater flexibility to achieve their career goals [20]. Companies like Google and Facebook, founded by individuals applying new technologies learned in higher education, exemplify this trend. Thus, understanding factors influencing entrepreneurial intentions in light of industry changes is crucial [21]. This study aims to explore the impact of entrepreneurial self-efficacy on the entrepreneurial intentions of business school students.

As students and their learning environments are the main focus of educational institutions, evaluating schools or departments from a student perspective can provide an important source of reference for educational effectiveness and reflection, while also complementing research on the internal differences in entrepreneurial intentions among business school students [22]. The Theory of Planned Behavior (TPB) is widely recognized for its explanatory power in decision-making behavior, particularly in entrepreneurial intentions and subsequent behaviors of new ventures [23]. Since student entrepreneurship often involves offering micro-entrepreneurial ideas, TPB is suitable for exploring this phenomenon [24]. First introduced by Ajzen and Fishbein in 1980, TPB posits that three independent variables—attitude, subjective norms, and perceived behavioral control—impact intentions, which in turn influence behavior [25]. Since its introduction, many scholars have engaged in TPB research, and while some have raised criticisms [26], much research supports TPB’s high explanatory power for human behavior, including entrepreneurial intentions and behaviors [27, 28]. Therefore, this study analyzes business school students, introduces TPB as the framework, and incorporates students’ sense of identification with their department as a moderating variable to expand TPB and investigate the influence of departmental identification on entrepreneurial intentions [22–24, 26, 27].

## 2. Literature review and hypotheses development

### 2.1. TPB and entrepreneurship

Entrepreneurial intentions are a key precursor to entrepreneurial behaviors, and understanding their formation is critical for promoting entrepreneurship among students. Nieuwenhuizen and Swanepoel [29] introduced the concept of "Personal Attitude (PA)–Attitude to becoming an entrepreneur," emphasizing that entrepreneurial intentions are driven by an individual’s willingness and readiness to engage in entrepreneurship. This concept aligns closely with entrepreneurial attitude, a construct from Ajzen’s Theory of Planned Behavior (TPB), which posits that attitudes, subjective norms, and perceived behavioral control collectively shape behavioral intentions [25]. In this framework, entrepreneurial attitude refers to an individual’s positive evaluation of entrepreneurial activities, while Personal Attitude (PA) highlights their readiness to act on those evaluations. Both constructs underline the significance of attitude as a foundation for entrepreneurial intentions. Accordingly, individuals with strong entrepreneurial attitudes are more likely to form intentions and take action [25].

Entrepreneurial intentions are shaped by various factors, including individual attributes such as personality traits, cognitive and non-cognitive skills, and contextual elements like education, cultural norms, and institutional support [30, 31]. Education and training programs, in particular, play a pivotal role by influencing students’ entrepreneurial attitudes and providing access to critical resources [32]. Recent studies have also emphasized the importance of integrating additional constructs, such as emotional intelligence and entrepreneurial identity, to enhance the explanatory power of TPB [33–35]. Entrepreneurial identity, for instance, highlights how individuals perceive themselves as entrepreneurs, often surpassing self-efficacy and perceived behavioral control in its impact on intentions [35]. By incorporating such perspectives, this study seeks to deepen the understanding of entrepreneurial attitudes and their role in shaping intentions, particularly within the context of higher education. The literature shows that entrepreneurial intentions are the best predictor of new venture behavior, highlighting the importance of intention models in understanding and predicting entrepreneurial activities [1, 3]. This suggests that studying the attitude to becoming an entrepreneur can help us better understand the formation process and influencing factors of entrepreneurial intentions.

Numerous studies have consistently identified entrepreneurial attitude as a fundamental antecedent of entrepreneurial intention, demonstrating its substantial influence on individuals’ career trajectories [33, 36]. Entrepreneurial attitude not only directly enhances the likelihood of engaging in entrepreneurial activities but also interacts with perceptions of barriers and support, shaping individuals’ career decisions [37]. Entrepreneurial self-efficacy has also been established as a critical determinant of entrepreneurial intention. For instance, Liñán and Chen [38] demonstrated that entrepreneurial attitude and self-efficacy significantly predict entrepreneurial intentions among university students in Spain and Taiwan, whereas environmental support did not exert a notable influence. Similarly, Amofah et al. [12], in a cross-national study involving MBA students from 32 countries, emphasized the strong positive effect of self-efficacy on entrepreneurial intentions, with prior experiences identified as a key predictor of self-efficacy. Miranda, Chamorro-Mera, Rubio, and Pérez-Mayo [39] further highlighted the role of gender as a moderating factor in entrepreneurial intention, underscoring the interplay between demographic variables and entrepreneurial determinants. Based on the findings from the literature reviewed [12, 28], it can be concluded that there is a positive relationship between entrepreneurial attitude and entrepreneurial intention. Therefore, the present study proposes the following hypothesis based on these recommendations:

H1: Business school students’ entrepreneurial attitude has a positive impact on entrepreneurial intentions.

Previous research has established a strong link between professional education and entrepreneurial self-efficacy, particularly among business school students [40]. Entrepreneurial self-efficacy, which reflects an individual’s confidence in performing entrepreneurial tasks, has been shown to positively correlate with entrepreneurial intentions. Professional management education enhances students’ self-efficacy by equipping them with entrepreneurial soft skills and fostering greater confidence in their abilities. For instance, students with exposure to entrepreneurship courses demonstrate higher levels of self-efficacy compared to their peers in other disciplines, such as organizational behavior or psychology [41]. Moreover, factors such as the presence of entrepreneurial role models, including friends and family members, and the number of management courses taken further reinforce students’ entrepreneurial self-efficacy, which in turn strengthens their entrepreneurial intentions.

Recent empirical studies have reinforced these findings, emphasizing the role of entrepreneurship education in enhancing self-efficacy and entrepreneurial intentions. For example, Lorz and Volery [41] found that entrepreneurship courses significantly improve students’ entrepreneurial self-efficacy, with this effect mediated by students’ attitudes toward entrepreneurship. Similarly, Bae et al. [42] demonstrated that entrepreneurship-focused programs instill a stronger belief in students’ abilities to succeed as entrepreneurs, a critical driver of entrepreneurial intentions. Using the TPB framework, Mejía et al. [28] highlighted how self-determination shapes entrepreneurial intention through its influence on entrepreneurial attitude and self-efficacy. Alam et al. [27], examining students at a Pakistani engineering university, found that entrepreneurial attitude and self-efficacy significantly predict entrepreneurial intentions, while social support showed no significant effect. These findings collectively underscore the pivotal role of entrepreneurship education in fostering self-efficacy and its subsequent impact on entrepreneurial intentions. Building on this literature, the present study proposes the following hypothesis:

H2: Business school students’ entrepreneurial self-efficacy has a positive impact on entrepreneurial intentions.

The Theory of Planned Behavior (TPB) has been widely applied as a framework for investigating entrepreneurial intentions [25, 43]. Within this framework, behavioral attitude is conceptualized as entrepreneurial attitude, subjective norms are represented as environmental support for entrepreneurship, and perceived behavioral control is often operationalized as entrepreneurial self-efficacy or entrepreneurial competence [36, 44]. Numerous studies have confirmed the significant and positive impact of entrepreneurial attitude and self-efficacy on entrepreneurial intentions, establishing these constructs as key determinants [27, 33, 35, 41]. However, the role of environmental support in shaping entrepreneurial intentions appears to be more context-dependent. While some studies suggest that environmental support positively influences entrepreneurial intentions by providing students with access to resources and fostering a culture of entrepreneurship, its effect is often weaker than that of entrepreneurial attitude and self-efficacy [35, 41]. This discrepancy highlights the importance of understanding how institutional environments, social norms, and resource availability collectively contribute to students’ entrepreneurial ambitions. Drawing on these findings, the present study posits that fostering a supportive social environment within university settings can play a crucial role in enhancing entrepreneurial intentions among business school students. Therefore, the following hypothesis is proposed:

H3: Entrepreneurial environmental support has a positive impact on the business school students’ entrepreneurial intentions.

### 2.2. The role of professional differentiation in the education of business schools

The differentiation of professional education knowledge can significantly impact the development of students’ core professional competencies and their identification with their department within the same college [45]. It is becoming increasingly clear that students from different academic disciplines have distinct departmental and professional identities that can significantly influence their career choices [40, 46]. This important area of research provides crucial insights into the role of department identification in shaping the career paths of university students.

Moreover, this research underscores the need to recognize the significant differences between students from different departments within the same college. To truly understand the factors that contribute to entrepreneurial intention or career choice among university students, it is essential to take a comparative perspective that recognizes the unique differences between departments [4, 47]. While this study cannot fully explain the underlying reasons for differences in students’ entrepreneurial intentions among different departments, it is clear that department identification plays a significant role in shaping students’ career choices [21, 48]. This highlights the importance of creating a supportive and inclusive academic environment that encourages students to explore their interests and passions, regardless of their academic discipline. In light of these findings, it is essential for higher education institutions to recognize the importance of department identification in shaping students’ career paths and to take active steps to support the unique needs and interests of students from different departments [18, 49]. By prioritizing diversity and inclusion, higher education institutions can help students develop a stronger sense of departmental and professional identity, thereby increasing their chances of success in the job market and beyond.

Identity, as a fundamental psychological construct, further elucidates how departmental and professional differentiation influence career outcomes. Social identity theory provides a framework for understanding how individuals define themselves through group membership, seeking a sense of belonging and aligning their behavior with group norms [43, 50]. Professional identity, a specific form of social identity, encompasses occupational commitment, job satisfaction, and a clear professional self-image [44]. Research demonstrates that individuals with stronger professional identities exhibit more positive attitudes toward their fields, greater interest in pursuing related careers, and higher evaluations of their own competencies [14, 51]. Additionally, professional and organizational identities are often interconnected, emphasizing the importance of fostering a sense of belonging within both academic and workplace contexts [1].

### 2.3. Moderating role of department identification

Department identification, a specific form of professional identity, reflects the extent to which students feel emotionally attached to and aligned with their academic departments. Research has shown that stronger departmental identification enhances students’ professional identity, which in turn positively influences their career choices and entrepreneurial intentions [3, 18]. This highlights the importance of creating academic environments that promote exploration, foster social connections, and develop entrepreneurial competencies [47]. Entrepreneurship education has been recognized as a critical factor in shaping professional identity and entrepreneurial intentions [21, 41, 42]. Studies demonstrate that entrepreneurial education enhances students’ entrepreneurial self-efficacy and fosters social identity, which collectively contribute to entrepreneurial aspirations [9, 21, 40]. For instance, Neneh [40] identified the interplay between social identity and entrepreneurial self-efficacy, while Lv et al. [21] and Liang et al. [9] highlighted the positive correlations between social capital, social identity, and entrepreneurial inclinations. These findings underscore the importance of integrating departmental identification into entrepreneurship education to strengthen students’ entrepreneurial intentions.

The Theory of Planned Behavior (TPB) [25] provides a robust framework for understanding entrepreneurial intentions. According to TPB, behavioral intention is determined by three key factors: attitude, subjective norms, and perceived behavioral control [25]. In entrepreneurial contexts, these constructs are commonly represented as entrepreneurial attitude, environmental support, and entrepreneurial self-efficacy, respectively [23, 27, 28, 33]. Numerous studies confirm that entrepreneurial intention is positively associated with professional identity [2, 51], social identity [14, 49], and self-efficacy [36, 52], while entrepreneurial education and social capital also play significant roles [7, 22, 41, 42, 48]. Demographic factors, such as gender [39, 45], academic major [18, 47], and cultural context [9, 12, 37], further shape entrepreneurial intentions. For example, professional identity influences work engagement among healthcare professionals [2], nursing students [44], and early childhood teachers. These contextual factors enrich the explanatory power of TPB by accounting for individual and institutional differences that influence entrepreneurial outcomes.

Numerous studies investigating entrepreneurial intention have relied on the Theory of Planned Behavior (TPB) [25, 43] and incorporated other variables to examine the direct or indirect influence of various factors on entrepreneurial intention. For instance, Sousa et al. [53] applied TPB to examine law students’ entrepreneurial intentions, using personality traits as moderating variables. Their findings indicated that traits such as extroversion and agreeableness significantly influenced entrepreneurial intentions. Similarly, Farida et al. [54] investigated vocational school students and found that entrepreneurship education moderated the impact of entrepreneurial attitude on intentions but did not diminish the effects of subjective norms or self-efficacy.

Research on identity’s impact on entrepreneurial intention frequently draws on Social Cognitive Theory [52], with TPB less commonly used for this purpose. However, Obschonka et al. [14] demonstrated that group identity moderates entrepreneurial intentions, showing that individuals with weaker group identity are influenced by external factors like social environments, while those with stronger identity are guided by internal attitudes and values. Ashforth et al. [1, 50] explored identity formation within organizations, proposing models that explain how professional and organizational identities influence individual behavior. These findings highlight the potential of integrating identity constructs into TPB to deepen our understanding of entrepreneurial intentions.

In addition to personality and identity, other factors have also been found to influence entrepreneurial intention. For example, entrepreneurial education has been found to have a positive impact on entrepreneurial intention [7, 22, 41, 42]. The impact of social capital on entrepreneurial intention has also been investigated, with some studies finding a positive correlation [9, 48], while others finding that the relationship is more complex and context-dependent [32]. Finally, professional identity has been found to influence work engagement among healthcare professionals [2], nursing students [44], and early childhood teachers. Based on the analysis of the above literature, this study proposes the following hypotheses:

H4: Business school students’ department identification positively moderate the relationship between entrepreneurial attitudes and intentions.
H5: Business school students’ department identification positively the relationship between entrepreneurial self-efficacy and intentions.
H6: Business school students’ department identification positively moderate the relationship between entrepreneurial environmental support and intentions.

**Fig 1:**
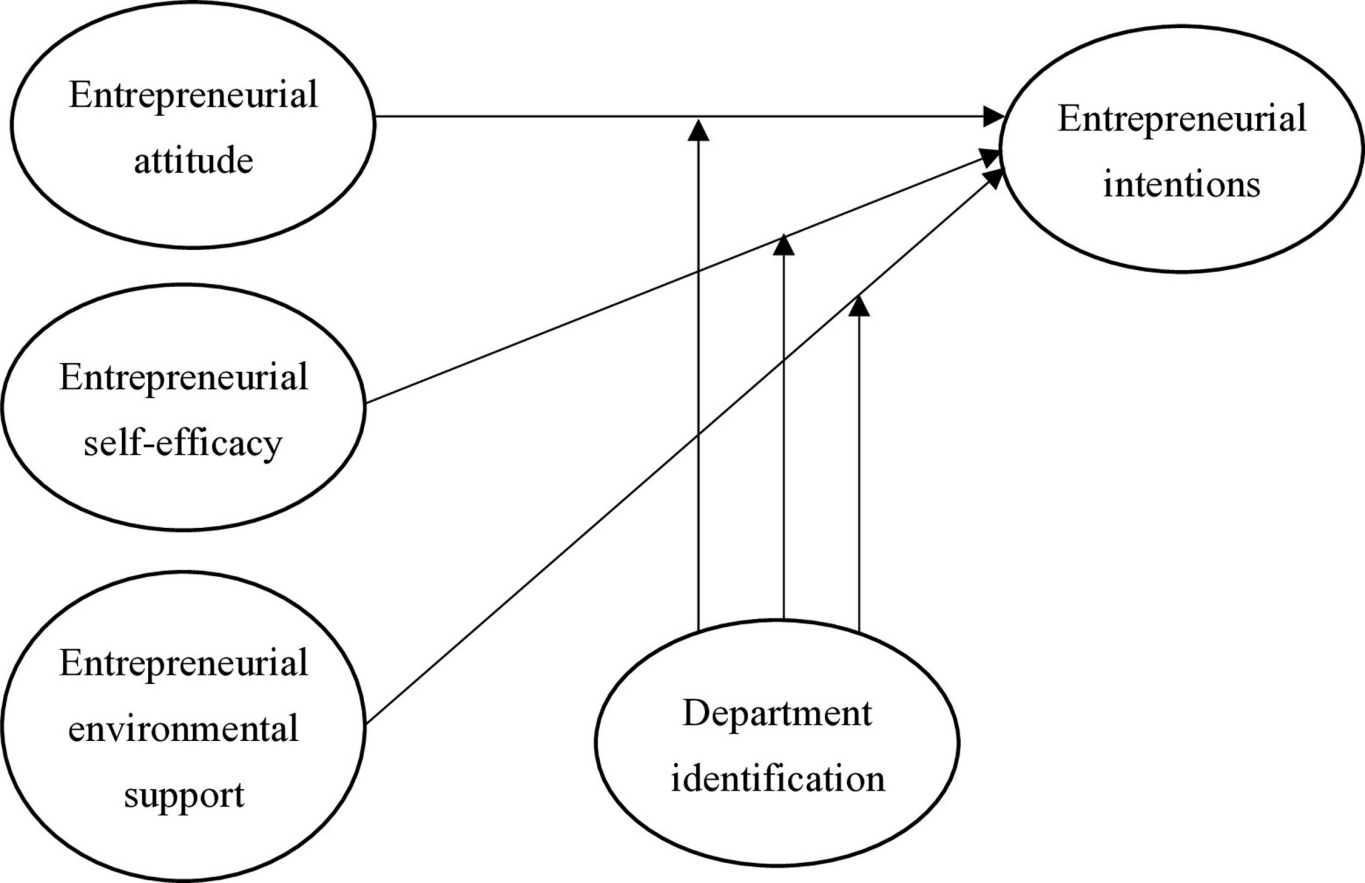
Research framework. This figure illustrates the proposed research framework, highlighting the relationships between entrepreneurial attitude, entrepreneurial self-efficacy, entrepreneurial environmental support, and entrepreneurial intentions, as well as the moderating role of department identification.

Based on the arguments above, we develop our research model as shown in Fig 1.

## 3. Methodology

### 3.1. Sampling

The study was conducted in several public universities in the eastern provinces of Mainland China, selecting and merging three fields of study: Business Administration, International Business, and Accounting and Financial Finance. The study focused on business and finance students for several reasons. Firstly, these students are typically more exposed to entrepreneurship education and resources, making them an ideal group to study the impact of attitudes, self-efficacy, and environmental support on entrepreneurial intentions. Studies have shown that entrepreneurship education can significantly influence students’ entrepreneurial intentions by enhancing their knowledge, skills, and attitudes towards entrepreneurship [42, 55]. This selection allows for a deeper understanding of how these factors interact within a context where entrepreneurship is often encouraged and supported. Secondly, business and finance students are likely to have more immediate access to entrepreneurial opportunities and networks, which can significantly influence their entrepreneurial behavior. Research indicates that access to entrepreneurial networks and resources plays a crucial role in shaping entrepreneurial intentions and behaviors [56, 57]. By focusing on this group, the study can provide more targeted insights and practical implications for educational institutions and policymakers working within similar environments.

The study employed a linear structural equation modeling (SEM) approach to analyze the relationships between entrepreneurial attitudes, entrepreneurial self-efficacy, entrepreneurial environmental support, academic identity, and entrepreneurial intentions, aiming to construct causal relationships between the main variables. Partial Least Squares Structural Equation Modeling (PLS-SEM) was used to estimate the model coefficients and related testing indicators to measure the model’s fitness, including basic fitness, overall model fitness, and internal structural fitness. The study utilized purposive sampling and was conducted from September 1 to November 31, 2022, collecting a total of 1,632 valid questionnaires. Among the participants, 688 students were from the Business Administration department, 623 were from the Accounting and Financial Finance department, and 312 were from the International Business department. In terms of geographical location, a higher proportion of respondents were from northern universities (51.2%) compared to central (29.5%) and southern universities (19.3%). The sample was roughly balanced in terms of gender, with 49.0% of the respondents being male and 51.0% female. A relatively small percentage of respondents came from low-income families (17.3%), with the majority being from well-off families (84.2%), and only 1.7% from high-income families. About a quarter of the respondents (25.5%) had applied for a grant.

### 3.2. Measures

This study employs a structured questionnaire to measure entrepreneurial attitudes, entrepreneurial self-efficacy, entrepreneurial environmental support, entrepreneurial intentions, and departmental identification. The measurement scales for entrepreneurial attitudes, entrepreneurial self-efficacy, entrepreneurial environmental support, and entrepreneurial intentions were adapted from Nieuwenhuizen and Swanepoel [29]. Meanwhile, departmental identification was measured using a scale developed by Pretorius and Padmanabhanunni [51]. These scales were chosen for their theoretical robustness and previous applications in entrepreneurial research. All items were rated on a seven-point Likert scale, ranging from 1 ("strongly disagree") to 7 ("strongly agree").

The original study by Nieuwenhuizen and Swanepoel [29] focused on cross-national comparisons of entrepreneurial constructs among business school students in South Africa and Poland, examining levels and differences across these variables. In this study, the scales were adapted to investigate the relationships among entrepreneurial attitudes, self-efficacy, environmental support, and intentions, with a specific focus on the moderating role of departmental identification. The contextual adaptation involved ensuring that the scales were applicable to Asian business school students while retaining their theoretical integrity. The contents of each dimension are explained as follows:

Entrepreneurial attitude refers to the extent to which students positively evaluate entrepreneurship as a career option, encompassing their interest, satisfaction, and perceived benefits. This construct was measured using five items adapted from Nieuwenhuizen and Swanepoel [29].

Entrepreneurial self-efficacy is defined as an individual’s confidence in their ability to successfully perform entrepreneurial tasks, such as identifying opportunities, managing resources, and overcoming challenges. This construct was assessed using 12 items adapted from Nieuwenhuizen and Swanepoel [29], tailored to reflect students’ contexts.

Entrepreneurial environmental support captures students’ perceptions of supportiveness within their immediate environment, including family, peers, and cultural context, for pursuing entrepreneurship. This dimension was measured using five items, also adapted from Nieuwenhuizen and Swanepoel [29].

Departmental identification refers to students’ emotional connection and sense of belonging to their academic departments. It was measured using four items adapted from the identity scale proposed by Pretorius and Padmanabhanunni [51].

Entrepreneurial intentions represent students’ deliberate plans and determination to start a business in the future. This dimension was assessed using four items adapted from Nieuwenhuizen and Swanepoel [29].

To ensure the validity and applicability of the instrument for the target population, this study conducted a content validity assessment and a pilot test prior to the main study. Expert evaluations were conducted during the content validity assessment, where entrepreneurship researchers and educators reviewed the items for clarity, relevance, and contextual appropriateness. Based on their feedback, minor revisions were made to improve item phrasing and ensure alignment with the study’s objectives. The pilot test was conducted with 30 business school students, who provided additional feedback on the clarity and comprehensibility of the items. Their responses were analyzed to confirm the reliability and applicability of the questionnaire items, and adjustments were made to enhance their relevance to a student context. Further details of the specific questionnaire items are provided in S1 Appendix, enabling readers to access the full scope of the instrument.

### 3.3. Ethical statement

The current study involved human participants and was approved by the Ethics Review Committee of Foshan University of Foshan University in Foshan, China, following a thorough review to ensure ethical compliance. The research was carried out in line with the ethical standards established by the Ethics Review Committee of Foshan University. All participants were fully briefed about the nature of the research, including its potential benefits and risks. They were also informed about their freedom to discontinue participation at any time. Verbal informed consent was obtained from each participant prior to their involvement in the study.

## 4. Analysis and results

### 4.1. Measurement

The questionnaire’s reliability and validity are displayed in Tables 1 and 2. The dependability of all items was demonstrated to be high, as indicated by the Cronbach’s alpha coefficient, which surpassed the threshold of 0.7. The Composite Reliability Coefficient exhibited a similarly elevated level, meeting the required threshold of 0.7 [58]. Furthermore, the factor loadings of each observation item above 0.7, indicating a high level of composite dependability. Consistent with the findings of Hair et al. [58], the Average Variance Extracted for each dimension in the investigation exceeded 0.5, indicating the presence of convergent validity. In addition, the cross-loadings for each dimension were lower than the factor loadings, and the correlation coefficient for each dimension was smaller than the square root of the Average Variance Extracted. This suggests that there is good discriminatory validity [58].

**Table 1:** Reliability and validity of all variables.

Latent constructs	AVE	CR	Cronbach’s alpha
Entrepreneurial Attitude (EA)	0.561	0.863	0.801
Entrepreneurial self-efficacy (ESE)	0.506	0.902	0.877
Entrepreneurial Environmental Support (EES)	0.770	0.943	0.925
Department identification (DI)	0.681	0.895	0.843
Entrepreneurial Intentions (EI)	0.707	0.923	0.896

**Table 2:** Discriminant validity.

	1	2	3	4	5
1. Entrepreneurial Attitude	0.749				
2. Entrepreneurial self-efficacy	0.670	0.712			
3. Entrepreneurial Environmental Support	0.512	0.629	0.877		
4. Department identification	0.548	0.684	0.812	0.825	
5. Entrepreneurial Intentions	0.496	0.655	0.701	0.778	0.841

### 4.2. Hypotheses testing

The results presented in the table show the coefficients (β value), t-value, p-value, effect size (f^2^) and the outcome of the statistical tests for each of the hypotheses tested in the study. The principal method utilized for data analysis in this inquiry is Partial Least Squares-SEM. The overall model fit is assessed using the Stone-Geisser-Criterion (Q^2^), coefficient of determination (R^2^), and standardized root mean square residuals (SRMR). The standardized root mean square residual (SRMR) had a value below 0.08, indicating a good fit. The Q^2^ values were greater than 0, indicating a positive predictive ability. The R^2^ values were highly significant, with a threshold of 0.20.

**Fig 2:**
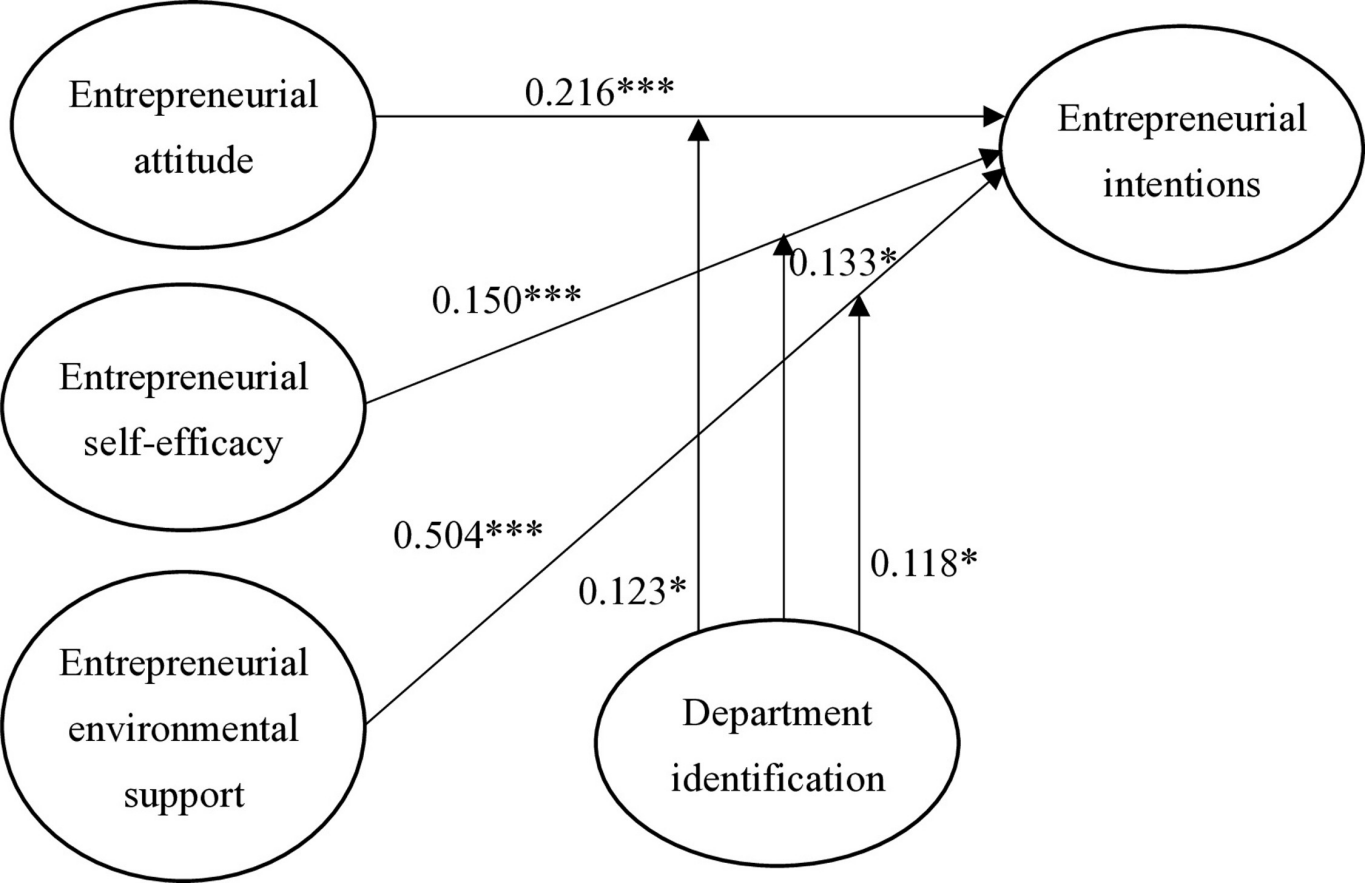
Inner structural model. This figure displays the inner structural model derived from Partial Least Squares Structural Equation Modeling (PLS-SEM) analysis, showing standardized path coefficients and their significance levels for the relationships between key constructs.

As results shown in Table 3 and Fig 2, H1 proposed a positive relationship between entrepreneurial attitude and entrepreneurial intentions, and the study results support this relationship (β = 0.216, t-value = 9.191, p < 0.001, f^2^ = 0.021). This suggests that individuals with a strong entrepreneurial attitude are likely to exhibit higher entrepreneurial intentions. H2 proposed a positive relationship between entrepreneurial self-efficacy and entrepreneurial intentions, and the study findings provide support for this relationship (β = 0.150, t-value = 5.933, p < 0.001, f^2^ = 0.079). This indicates that individuals who possess high levels of entrepreneurial self-efficacy are likely to exhibit higher levels of entrepreneurial intentions. H3 proposed a positive relationship between entrepreneurial environmental support and entrepreneurial intentions, and the study results strongly support this relationship (β = 0.504, t-value = 18.763, p < 0.001, f^2^ = 0.764). This suggests that individuals who possess high levels of entrepreneurial environmental support are likely to exhibit higher levels of entrepreneurial intentions.

**Table 3:** Results of hypotheses testing.

Path	β value	t-value	p-value	f^2^	Results
H1: EA -> EI	0.216	9.191	< 0.001	0.021	Support
H2: ESE -> EI	0.150	5.933	< 0.001	0.079	Support
H3: EES -> EI	0.504	18.763	< 0.001	0.764	Support
H4: EA * DI -> EI	0.123	1.759	< 0.05	0.103	Support
H5: ESE * DI -> EI	0.133	1.732	< 0.05	0.093	Support
H6: EES* DI -> EI	0.118	1.683	< 0.05	0.085	Support

H4, H5 and H6 proposed that department identification moderates the relationships between entrepreneurial attitude and entrepreneurial intentions (β = 0.123, t-value = 1.759, p < 0.05), entrepreneurial self-efficacy and entrepreneurial intentions (β = 0.133, t-value = 1.732, p < 0.05), and entrepreneurial environmental support and entrepreneurial intentions (β = 0.118, t-value = 1.683, p < 0.05), respectively. The study results provide support for all of these hypotheses, with significant interaction effects between each independent variable and department identification. The effect sizes for the interaction effects range from small to moderate, with f^2^ values of 0.103, 0.093, and 0.085, respectively.

## 5. Conclusions

### 5.1. Discussion

These findings indicate that persons who have a favorable disposition towards entrepreneurship, possess strong self-efficacy beliefs in their capacity to initiate and manage a business, and receive substantial support from their surroundings are more likely to have elevated levels of entrepreneurial ambitions. This study provides a comprehensive framework that integrates entrepreneurial attitude, self-efficacy, and environmental support, while uniquely highlighting their collective influence on entrepreneurial intentions. Such an integrated approach bridges gaps in previous research, which often treated these factors in isolation. By integrating these factors, the study provides a more nuanced understanding of entrepreneurial intentions, which has not been fully explored in previous studies. The inclusion of departmental identification as a moderating variable further distinguishes this work by uncovering its amplifying effects on these relationships, offering both theoretical insights and practical implications.

This is important information for business school students who may be interested in starting their own businesses. The findings emphasize the importance of having a positive mindset, strong self-belief, and a supportive environment in achieving their entrepreneurial goals. Additionally, the study shows that department identification, or the extent to which individuals identify with their academic department or discipline, can moderate the relationships between the independent variables and entrepreneurial intentions. This moderation highlights how students’ identification with their academic environment can enhance their entrepreneurial motivations, suggesting that fostering stronger departmental connections can lead to more effective educational strategies for entrepreneurship. These insights provide actionable guidance for business school students, educators, and policymakers to foster a supportive entrepreneurial ecosystem within academic institutions.

The finding that there is a positive relationship between entrepreneurial attitude and entrepreneurial intentions is consistent with previous research [38]. As the theory of planned behavior proposes, attitudes are a critical determinant of intentions, which in turn guide behavior [25]. This study makes an important theoretical contribution by extending the theory of planned behavior, demonstrating empirically how a positive entrepreneurial attitude can act as a foundational driver of entrepreneurial intentions in the context of higher education. This study extends the theory of planned behavior by empirically demonstrating that a positive entrepreneurial attitude is a robust predictor of entrepreneurial intentions among business school students, reinforcing the theoretical framework with new empirical evidence. This suggests that individuals who possess a positive attitude towards entrepreneurship are more likely to exhibit higher levels of entrepreneurial intentions. Unlike previous research, this study explicitly connects entrepreneurial attitudes to practical implications, such as shaping entrepreneurship curricula that foster positive attitudes, thus making its findings actionable for educators and administrators.

Moreover, the consistency of this finding with previous research [38] supports the notion that attitudes are a robust predictor of intentions. By confirming and extending these findings, this study highlights the importance of developing and nurturing a positive attitude towards entrepreneurship in educational settings, thus contributing to a deeper understanding of the mechanisms underlying entrepreneurial intentions. According to Bandura’s social cognitive theory, self-efficacy beliefs play a crucial role in determining behavior. The findings of this study align with the proposed theory, since it revealed a direct correlation between entrepreneurial self-efficacy and entrepreneurial goals. Our findings advance Bandura’s framework by emphasizing how entrepreneurial self-efficacy not only predicts entrepreneurial intentions but also serves as a pivotal mechanism through which educational interventions can influence students’ entrepreneurial outcomes. This study contributes to the literature by providing empirical support for the role of entrepreneurial self-efficacy in shaping entrepreneurial intentions, specifically within the context of business school students. This extends the applicability of Bandura’s theory to the field of entrepreneurship education. This suggests that persons with strong self-efficacy views in their capacity to initiate and manage a firm are more inclined to have greater levels of entrepreneurial inclinations. The consistency of this finding with Sharahiley [33] and Newman et al. [16] that self-efficacy is a robust predictor of entrepreneurial intentions. These insights emphasize the importance of designing entrepreneurship education programs that prioritize the development of self-efficacy, equipping students with the confidence and skills required to pursue entrepreneurial endeavors.

The finding that there is a positive relationship between entrepreneurial environmental support and entrepreneurial intentions is not only supported by the present study, but also by numerous scholars who suggest that creating an entrepreneurial-friendly environment is crucial in fostering entrepreneurship [7, 59]. This study builds upon these findings by contextualizing entrepreneurial environmental support within higher education institutions, demonstrating its significant impact on business school students. Moreover, an entrepreneurial-friendly environment can signal to individuals that entrepreneurship is a legitimate and valuable pursuit, thus positively influencing their intentions to pursue entrepreneurial activities [23]. It can also provide access to financial resources and supportive policies and regulations, which are essential for the growth and development of entrepreneurial activities. This contribution is particularly timely given the increasing emphasis on entrepreneurship as a viable career path, highlighting the need for institutions to create environments that actively promote and sustain entrepreneurial efforts. By empirically validating these relationships, the study provides actionable insights for policymakers and educational institutions to design environments that effectively support and encourage entrepreneurial pursuits among students.

When discussing the results of this study, it is important to consider the theoretical implications of the finding that department identification moderates the relationships between entrepreneurial attitude, entrepreneurial self-efficacy, entrepreneurial environmental support, and entrepreneurial intentions. This finding is consistent with several publications in entrepreneurship research, including Guo et al. [5], and Shirokova et al. [32]. The moderating role of department identification represents a novel theoretical contribution, as it connects social identity theory with entrepreneurial intention models. This integrated perspective advances our understanding of how contextual and identity-related factors interact to shape entrepreneurial behavior. This study advances the understanding of the moderating role of department identification by providing empirical evidence that highlights its significant impact on entrepreneurial intentions. This idea is supported by previous research [60], which has shown that an individual’s social identity, represented by their identification with a particular group or community, can influence their entrepreneurial intentions. The importance of social identity and institutional context in shaping entrepreneurial behavior and outcomes has also been noted by other studies [7, 61]. Our findings emphasize the strategic role of fostering departmental identification within educational institutions, suggesting that strengthening students’ ties to their academic departments can serve as a critical lever to amplify entrepreneurial motivations and outcomes.

By taking into account the role of social identity in the entrepreneurial process, policymakers and organizations can create a supportive environment that is responsive to the social identity and institutional context of individuals, thereby increasing the likelihood of individuals pursuing entrepreneurial activities [59]. In sum, this study contributes to the literature by providing both theoretical advancements and practical strategies for cultivating entrepreneurial intentions through enhanced departmental identification, self-efficacy, and supportive environments, laying a robust foundation for future research and application in entrepreneurship education.

### 5.2. Practical implications

According to the Theory of Planned Behavior, the present findings suggest that individuals’ attitudes, self-efficacy beliefs, and perceived social norms are crucial determinants of their entrepreneurial intentions. Cultivating a positive attitude towards entrepreneurship, building strong self-belief, and establishing a supportive network are key factors in enhancing entrepreneurial intentions and success. These findings have significant implications for aspiring entrepreneurs, as they can utilize this knowledge to increase their chances of success by adopting these advantageous attitudes, beliefs, and environmental support.

For business school students, these results are particularly relevant, as they highlight the critical role of attitudes, beliefs, and environmental factors in shaping entrepreneurial intentions. According to the Theory of Planned Behavior, these factors are essential precursors to entrepreneurial actions, such as starting a business. Students who integrate these elements into their entrepreneurial endeavors are likely to have higher levels of entrepreneurial intentions, which can lead to increased success as business owners.

Moreover, the findings suggest that department identification can moderate the relationship between the independent variables and entrepreneurial intentions. This underscores the importance of considering the unique characteristics and resources of different academic departments or disciplines when promoting entrepreneurship. Aspiring entrepreneurs should tailor their approach to their respective fields to effectively leverage the available resources and overcome potential obstacles.

In conclusion, the present study provides compelling evidence for the importance of attitudes, beliefs, and environmental factors in shaping entrepreneurial intentions and success. By adopting a positive mindset, building self-belief, and establishing a supportive network, business school students can enhance their entrepreneurial intentions and increase their likelihood of future success as entrepreneurs. The insights from this study are relevant to business school students, policymakers, and career counselors, and can guide the development of effective interventions to foster entrepreneurial behavior.

### 5.3. Limitations

This study offers valuable insights into the factors shaping entrepreneurial intentions; however, several limitations should be acknowledged, alongside corresponding directions for future research. First, while entrepreneurial attitudes, self-efficacy, and environmental support are critical factors influencing entrepreneurial intentions, this study does not fully address the broader entrepreneurial process. For example, barriers such as access to financial resources, regulatory challenges, and personal resilience may also significantly influence entrepreneurial success. Future research should explore these additional determinants, particularly those that impact the transition from entrepreneurial intentions to tangible outcomes, such as business establishment and long-term success. By examining the interplay between intentions and actual entrepreneurial behavior, future studies could provide a more comprehensive understanding of the entrepreneurial journey.

Second, the research sample consisted of students from Business Administration, International Business, and Accounting and Financial Finance programs at public universities in the eastern provinces of Mainland China. This specific focus limits the generalizability of findings to other academic disciplines and geographic regions. Future research should broaden its scope by including students from diverse disciplines, such as engineering, ICT, and marketing, to compare how different academic environments shape entrepreneurial intentions. Comparative studies across regions and cultures could also uncover how local norms, institutional frameworks, and regional support systems influence entrepreneurial behavior, providing a richer understanding of cross-cultural entrepreneurial dynamics.

Third, the study relied on self-reported data, which is susceptible to biases such as social desirability and recall inaccuracies. To mitigate these issues, future research could employ alternative methods, such as behavioral observations, longitudinal studies, or experimental designs, to capture more objective and reliable measures of entrepreneurial tendencies. Incorporating mixed-method approaches that combine quantitative and qualitative data could further enhance the robustness and depth of future findings, enabling a more nuanced understanding of entrepreneurial phenomena.

Finally, while this study examines the moderating role of departmental identification, it does not account for other contextual factors that may influence entrepreneurial intentions, such as family entrepreneurial background, peer networks, or personal values. Future research could explore how these additional contextual variables interact with entrepreneurial attitudes, self-efficacy, and environmental support. For example, analyzing the role of family entrepreneurial history could provide insights into intergenerational influences on entrepreneurial behavior, while examining the dynamics of peer groups might reveal how social norms and shared values shape entrepreneurial decisions. By integrating these contextual factors, future research could offer targeted recommendations for educators and policymakers to design more effective interventions that foster entrepreneurial activity.

By addressing these limitations, future studies could significantly advance the understanding of entrepreneurial intentions and their determinants, contributing to more effective strategies for promoting entrepreneurship in various academic, cultural, and institutional settings.

## Supporting information

S1 Appendix (DOCX)
