# Supplementary material for: Exploring factors influencing university students’ entrepreneurial intentions: The role of attitudes, beliefs, and environmental support
Source: PLoS One. 2025 Jan 9;20(1):e0316392. doi: 10.1371/journal.pone.0316392 (PMC11717231; doi:10.1371/journal.pone.0316392)
Supplement: S1 Appendix — (DOCX) [file pone.0316392.s001.docx]

Appendix 1

| Variables | Items | Reference |
| --- | --- | --- |
| Entrepreneurial Attitude | Entrepreneurship has more benefits than drawbacks for me | Nieuwenhuizen and Swanepoel [29] |
|  | Entrepreneurship is very attractive to me |  |
|  | Compared to other career options, I prefer entrepreneurship |  |
|  | Entrepreneurship brings me great satisfaction |  |
|  | Past experiences have made a very positive contribution to my entrepreneurial attitude |  |
| Entrepreneurial self-efficacy | I believe I can effectively lead and manage a team |  |
|  | I am confident in handling daily challenges and crises |  |
|  | I foster an innovative environment |  |
|  | I persist in the face of adversity |  |
|  | I can identify new product or service opportunities |  |
|  | I can set appropriate prices for new products or services |  |
|  | I can develop effective marketing strategies |  |
|  | I am good at communicating information |  |
|  | I build strong relationships with potential mentors and advisors |  |
|  | I can collaborate effectively with team members to achieve common goals |  |
|  | I am skilled in negotiating and persuading stakeholders |  |
|  | I am willing to make sacrifices to start the entrepreneurial process |  |
| Entrepreneurial Environmental Support | My classmates approve of my entrepreneurial decision |  |
|  | My family considers entrepreneurship superior to other job options |  |
|  | My family supports my entrepreneurial decision |  |
|  | My family can provide assistance for my entrepreneurship |  |
|  | My country's culture is very conducive to entrepreneurial activities |  |
| Entrepreneurial Intentions | I have no doubt that I will start a business in the future |  |
|  | I have carefully considered starting a business in the future |  |
|  | I have a strong intention to start a business in the future |  |
|  | Past experiences have significantly contributed to my entrepreneurial intentions |  |
| Department identification | The field of study I am pursuing is an industry with ongoing development | Pretorius and Padmanabhanunni [51] |
|  | There are unlimited opportunities in the relevant fields of study |  |
|  | The success of the relevant industries of my field of study is important for the future economic development of our country |  |
|  | Compared to non-related jobs, the salaries for the relevant field of study are generally higher |  |
